# Impact of COVID-19 on food security and diet quality in Chilanga District, Zambia

**DOI:** 10.1186/s41043-024-00523-5

**Published:** 2024-02-15

**Authors:** Shela Sridhar, Janella Kang, Joyce Makasa, Sally Bell-Cross, Isabel Madzorera, Ethan Zulu, Davidson H. Hamer

**Affiliations:** 1 Division of Global Health Equity, Brigham and Women’s Hospital, Boston, MA USA; 2 Department of Child, Youth, and Family Studies, University of Nebraska – Lincoln, Lincoln, NE USA; 3 I4life, Lusaka, Zambia; 4 Division of Community Health Sciences, School of Public Health, University of California, Berkeley, Berkeley, CA USA; 5 Right to Care, Lusaka, Zambia; 6 Department of Global Health, Boston University School of Public Health, Boston, MA USA

**Keywords:** Diet diversity, Nutrition, Prime diet quality score, Minimum dietary diversity-women score

## Abstract

**Introduction:**

Food security and nutrition have been severely impacted during the COVID-19 pandemic, particularly in low- and middle-income countries (LMICs). We aimed to quantify the impacts of the pandemic on food security and diet diversity within Chilanga District in Zambia and identify target areas for high-impact social protection and safety net programs.

**Methods:**

We conducted a cross-sectional study in Chilanga district immediately after the Omicron variant surge in February 2022. Diet quality and food security were assessed based on a household diet questionnaire and a Minimum Dietary Diversity-Women (MDD-W) score was calculated. A paired t-test was used to determine whether there was a statistically significant change in the MDD-W score and McNemar test was used to investigate the change in food security between the pre- and peri-COVID-19 period.

**Results:**

Compared to the pre-COVID-19 period, there were increases in food prices across the board in the peri-COVID-19 period and decreased consumption of key food categories including legumes, dairy and vitamin A rich foods. Despite high rates of food insecurity, only 6.6% of surveyed households received any cash or in-kind assistance from a government agency, non-profit, or other organization in the post-COVID-19 period.

**Conclusion:**

The COVID-19 pandemic had significant impacts on food security and dietary diversity in Chilanga district. This is particularly relevant in the low-income communities that we surveyed, which had pre-existing challenges with food security. Additional resources must be invested in Chilanga District and similarly affected areas to address this gap in access to food and promote national equity.

*Trial Registration* N/A.

## Introduction

The COVID-19 pandemic has had a notable impact on food security and the nutritional well-being of many populations around the world [1]. This adverse impact has been in large part due to public health measures implemented across the globe at the start of the pandemic which were intended to curb the spread of COVID-19 [2, 3]. While these public health measures were critical in the containment of the disease and given the severity of symptoms and long-term health effects of SARS-CoV-2 [4, 5], the side effects of the lockdowns included economic, mental health, education, and food security challenges particularly in adolescent populations across sub-Saharan Africa [3, 6]. These measures also had a dramatic effect at the community level, often with the most vulnerable populations being differentially affected [1]. This global decline in food security has ultimately diminished the nutritional status of communities as food security is closely linked with nutritional security, particularly in regard to quantity and quality of food [7]. While food security alone is not enough to ensure nutritional security of a community, this is a fundamental necessity to promote adequate nutrition of children, adolescents, and families. Given this, the dramatic impact of the COVID-19 pandemic must be adequately quantified to ensure that funds are properly distributed to those communities with the greatest need as we move out of the pandemic and into a “new normal” [8].

Since 2019, the number of individuals around the world experiencing acute food insecurity increased from 135 to 345 million in 2022. Much of this increase can be attributed to the economic impact of the COVID-19 pandemic [9, 10]. In Zambia, 1.35 million people were described as food insecure between July 2022 and September 2022 [11], and this number rose to 1.59 million by September 2023 with the number expected to rise to 2.04 million by 2024 [12]. Currently, the United States Agency for International Development (USAID) ranks Zambia 102 out of 113 countries around the world on the Global Food Security Index, which takes into account the affordability and availability of food as well as the quality, safety, and sustainability of food systems [13]. The Integrated Food Security Phase Classification (IPC) classifies food insecurity across 5 phases (phase 1 being minimal food insecurity and phase 5 being catastrophic) [11]. In Lusaka Province, between July to September 2021, 50% of the population was identified as being at phase 2, or stressed (or chronically food insecure), and 6% at phase 3, experiencing acute food and livelihood crisis [11].

Much of the rise in food insecurity over the last 2 years can be attributed to reduced livelihoods due to COVID-19 as well as global macroeconomic instability [11]. In many LMICs, smaller and medium size enterprises play significant role in food supply chains and these businesses were among the most negatively impacted by the pandemic [14]. In one Peruvian study, 98% of respondents reported that there were adverse economic impacts due to the pandemic and just under 50% of households were at risk for moderate or severe household food security [15]. In Ethiopia, one study noted reduce-in person support, disruptions in social protection programs, increased food prices and income losses, all associated with decreases in diet diversity and food security [16].

The impact of this acute food insecurity will have long-term and far-reaching impacts on the health and well-being of communities, particularly marginalized and vulnerable populations. Multiple studies have linked food insecurity with poor health outcomes including losses to follow-up care for chronic conditions such as HIV [17], as well as higher rates of malnutrition [18] which may lead to increased rates of hospitalization, prolonged hospitalization, and poor recovery for children and adults alike [10].

This cascade affects the most vulnerable populations including children under 5 years. Since the start of the COVID-19 pandemic, it has been estimated that the prevalence of malnutrition in under-five children has increased by 14.3% in low- and middle-income countries (LMICs) [10]. This places further burdens on already overwhelmed health care systems in these countries further exacerbated by increases in poverty and decreased access to care [10].

Understanding the impact of the COVID-19 pandemic on food security within a community is the first step to identifying where and how to invest in social protection and safety net programs. There was a reduced income for many who were informally, or even formally employed resulting in reduced supply at the macroeconomic level as well as decreased individual income to purchase food [3, 14, 16]. While there was no true shutdown after the Omicron surge when our study was conducted, we hypothesized that these impacts had a continued effect on food security and diet diversity.

Many of the studies describing the decline in food security during the COVID-19 pandemic are based on macroeconomic and country-level data. However, our study aimed to describe these impacts on Chilanga district specifically, with a focus on the prices of key food groups and dietary diversity using the Minimum Dietary Diversity-Women (MDD-W) score for households during this crisis period. Our study was conducted immediately after the Omicron surge in January 2022. We selected this smaller community due to pre-existing research capacity and data collection infrastructure and because there was a significant population vulnerable to undernutrition due to known inadequate resources and several families experiencing social and economic inequity. By understanding this smaller community, more targeted interventions can be considered. While larger macroeconomic interventions are required at the national level, individual communities can facilitate change at a grassroots level.

## Methods

We conducted a cross-sectional study of 393 households in Chilanga district, Lusaka Province in Zambia. Written informed consent was obtained for each household and translated into the local language, either Nyanja or Bemba. In each household, a household questionnaire designed to assess demographic and dietary habits was completed by one member of the household. Anthropometric data for children and adolescents was collected as well, these data are reported elsewhere [19].

### Study site and population

This study was conducted in 4 compounds (neighborhoods) within Chilanga district, Freedom, Kuzimbva, Linda, and Mwembeshi. The district has a population of just over 107,000 individuals, with more than 50% under the age of 19 years. About 20% of the population is designated as urban with the remaining 80% designated as rural [20]. The survey was executed through i4life, a local, nutrition-focused non-governmental organization (NGO). Right to care provided logistical support. The study population included any household with children with at least half of the recruited households required to have at least 1 adolescent (defined as under age 19 years) in the household. The adolescent criterion was included to identify adolescent malnutrition; full results are presented elsewhere [19]. Exclusion criteria included any households without children or adolescents or which included members of the data collectors’ family.

### Sampling

Data collectors were fluent in the local language and had previous surveying experience. Regions within compounds were identified through purposive sampling based on the data collectors’ knowledge of the communities. They identified the streets and areas in each compound a priori. Once data collectors were within each compound, a systematic randomized sample was used and data collectors identified every third house within a neighborhood and collected anthropometric data from all children living in the household, as well, as a household-level diet questionnaire, answered by one member of the household which included information regarding household dietary practices from the pre-COVID-19 period (January 2019–January 2020) as well as current dietary practice about 2 years into the COVID-19 pandemic (February 2022). Local and district approvals were obtained for community engagement.

### Data management

Data were collected on tablets and input into REDCap (Research Electronic Data Capture, Vanderbilt University, Nashville, Tennessee, USA), a secure web application for building and managing online surveys and databases hosted at Brigham and Women’s Hospital [21]. Paper surveys were available in English and Nyanja for data collectors to use in case of technical challenges. Completed paper surveys were subsequently input into REDCap manually. No personal identifiers were collected.

The household diet questionnaire was adapted from a questionnaire developed by the Africa Research Implementation Science Education (ARISE) research group to evaluate the impact of COVID-19 on adolescent health in several sub-Saharan African countries [22]. Food lists were comprised of 20 food groups and adapted to the Zambian context. Diet quality was assessed based on the questionnaire and a Minimum Dietary Diversity-Women (MDD-W) score was calculated based on 10 food groups defined by the Food and Agriculture Organization (FAO) [23]. Foods were further categorized as unhealthy vs healthy based on the Prime Diet Quality Score (PDSQ) [24]. One member of each household was asked to complete the questionnaire. We asked participants how many times each type of food was consumed over the last 7 days. Each food that was consumed at least once during this period contributed to the MDD-W score. In addition to diet quality, the questionnaire included questions focused on food security such as skipping a meal and fear of running out of food as well as questions focused on local prices for food staples. Key demographic questions were obtained including the number of family members per household, age of head of household, employment status, household characteristics, education level, and household income.

At the completion of the household questionnaire, participants were provided with the opportunity to provide additional comments regarding the impact of COVID-19. We asked all participants “Is there anything else you would like to share with us regarding the impact of COVID-19 on your life or your ability to find food?” While not a formal qualitative research question, this question was used to identify additional challenges that our study team had not previously identified or considered and should be further investigated. This question was also used to understand potential local interventions that should be considered by the local team for advocacy purposes.

### Statistical analysis

Our primary outcome was the change in dietary diversity within each household which was measured using the MDD-W. Our secondary outcome was the changes in food security measured using a modified Food Insecurity Experience Scale [25]. Changes were measured based on the patterns in the seven days prior to interview (February 2022) for post-Covid measurements and between January 2019 and January 2020 for pre-Covid-19 measurements.

For categorical variables, frequencies and prevalence and means for continuous variables were used to summarize food security and change in diet quality. A paired t-test was used to determine whether there was a statistically significant change in MDD-W score between pre- and post-COVID-19. McNemar’s test was used to investigate the change in food security (worried about running out of food, skipped a meal, went without eating for a whole day, and received any assistance), between pre- and post-COVID-19 in Chilanga District. Statistical significance for both was determined as less than 0.05.

Data were analyzed with STATA 17.0 software. A significance level of *p* = 0.05 was used for all statistical tests and analyses.

## Results

We surveyed a total of 393 households across the 4 compounds. More than 50% of the households surveyed had a monthly household income of less than 1000 kwacha (46.2 USD based on the exchange rate at the time of the survey [xe.com]). At the time of the survey, 27.8% responded that they were unemployed (77.8% of respondents on behalf of the households were mothers), 27.6% were business owners, and 21.2% were casual workers. Almost half (46.5%) of respondents had completed some or all of secondary school (Table 1). Demographics across the compounds were similar (data not shown).

**Table 1:** Socio-demographic characteristics of study households

	*n*	%
Total households (*n* = 393)		
Number of family members in household	–	–
Presence of family member with HIV	77	19.6
Presence of family member with tuberculosis	17	4.4
Respondent (*n* = 391)		
Mother	304	77.8
Father	38	9.7
Grandparent	16	4.1
Child	19	4.9
Other	14	3.6
Monthly Household income level (*n* = 389)		
< 1000 kwacha^a^	216	55.5
1000–2400 kwacha	131	33.7
> 2500 kwacha	42	10.8
Occupation of respondent (*n* = 392)		
Unemployed	109	27.8
Business owner	108	27.6
Casual worker (maid or gardener)	83	21.2
Wage employment	52	13.3
Self-employed	35	8.9
Other	5	1.3
Education level of respondent (*n* = 389)		
None	23	5.9
Some/completed primary school education	171	44.0
Some/completed secondary/high school	181	46.5
Tertiary education	12	3.1
Other (literacy class, religious school)	2	0.5
*Water, sanitation, and hygiene*		
Access to clean water for washing hands (*n* = 392)	369	94.1
Access to clean water for cooking (*n* = 392)	365	93.1
Water source (*n* = 393)		
Borehole	113	28.8
Communal tap	86	21.9
Protected dug well	17	4.3
Tapped running water	155	39.4
Other	22	5.6
Soap in the household (*n* = 390)		
Yes	292	74.9
No	98	25.1

^a^21.49 kwacha = 1 USD as of Feb 4, 2022

### Food security

Most of the surveyed population (77.7%) were worried about running out of food in the last month while 64.1% had skipped a meal and almost a quarter (24.5%) had gone for a whole day without eating in the month preceding the survey (Table 2). We found a significant increase in the rates of food insecurity during the COVID-19 pandemic, compared to pre-COVID-19. In all categories, the OR were greater than 2. There was a significant increase in respondents who noted they were afraid of running out of food (OR 5.33, 95% CI 2.59–12.36, *p* < 0.001) when comparing peri-COVID-19 to pre-COVID-19 rates of worry. The OR of those who had skipped a meal pre- and post-covid was 4.17 (CI 2.19–8.60, *p* < 0.0001) (Table 3). This correlated with reported increases in food prices across the board, including for staples, pulses, fruit, vegetable, and animal sources (Table 4). In each category, greater than 95% of participants reported an increase in food prices. Despite these high rates of food insecurity, only 6.7% of the surveyed households received any cash or in-kind assistance from a government agency, non-profit, or other organization.

**Table 2:** Food security and price change pre-COVID-19 pandemic versus present (in the past month)

	In the past month		Pre-Covid	
	Total	%	Total	%
Worry about running out of food				
No	87	22.3	126	32.3
Yes	304	77.7	264	67.0
Skipped a meal				
No	141	35.9	179	45.5
Yes	252	64.1	214	54.5
Went without eating for a whole day				
No	296	75.5	316	80.4
Yes	96	24.5	77	19.6
Received any assistance				
No	365	93.4	368	94.8
Yes	26	6.6	20	5.2

**Table 3:** Food security by household

Variable	Odds ratio (OR)*	95% CI		*p* value
Worry about running out of food	5.33	2.59	12.36	< 0.0001
Skipped a meal	4.17	2.19	8.60	< 0.0001
Went without eating for a whole day	2.36	1.22	4.77	0.0079
Received any assistance	1.71	0.62	5.14	0.36

^*^Based on McNemar test with pre-Covid values as the reference

**Table 4:** Change in cost of food after Covid-19*

	Total	%
Staple price		
No change or decreased	11	2.9
Increased	374	97.1
Pulse price		
No change or decreased	11	2.8
Increased	378	97.2
Fruit price		
No change or decreased	9	2.3
Increased	375	97.7
Vegetable price		
No change or decreased	9	2.3
Increased	382	97.7
Animal source food price		
No change or decreased	9	2.3
Increased	383	97.7

*This is based on participant recall

### Diet diversity

When looking at the 20 categories of food in the questionnaire, cruciferous vegetables, other vitamin A rich fruits and vegetables, other whole fruits, poultry, red meat, fish and processed meat, consumption decreased by greater than 30% during the COVID-19 pandemic. There was a significant decrease of greater than 10% (*t*(393) = 9.139, *p* < 0.0001) in some “healthy” foods including vitamin A rich fruits and vegetables (other vitamin A rich fruits and vegetables remained stable) (Table 5). Additionally, consumption of other vegetables and whole citrus fruits as well as eggs increased greater than 10%. We also noted a stagnation and/or increase in “unhealthy” foods including refined grains and baked goods and vegetable oils. For instance, 91.5% of respondents noted that vegetable oil consumption remained the same or increased (Table 5).

**Table 5:** Change in diet quality between pre-Covid versus during Covid pandemic by compound*, *n* (%)

	Overall (across all four compounds)	**p value
Cruciferous vegetables		< 0.001
Decreased	127 (32.7)	
Same	237 (60.9)	
Increased	25 (6.4)	
Vitamin A rich dark green vegetables		0.094
Decreased	86 (22.1)	
Same	235 (60.4)	
Increased	68 (17.5)	
Other vitamin A rich fruits and vegetable		< 0.001
Decreased	143 (36.9)	
Same	177 (45.6)	
Increased	68 (17.5)	
Other vegetables		< 0.001
Decreased	111 (28.6)	
Same	216 (55.7)	
Increased	61 (15.7)	
Whole citrus fruits		< 0.001
Decreased	111 (28.7)	
Same	228 (58.9)	
Increased	48 (12.4)	
Other whole fruits		< 0.001
Decreased	122 (31.5)	
Same	230 (59.4)	
Increased	35 (9.0)	
Poultry		< 0.001
Decreased	122 (31.6)	
Same	246 (63.7)	
Increased	18 (4.7)	
Red meat		< 0.001
Decreased	128 (33.4)	
Same	236 (61.6)	
Increased	19 (5.0)	
Fish		< 0.001
Decreased	135 (35.0)	
Same	221 (57.3)	
Increased	30 (7.8)	
Processed meat		< 0.001
Decreased	118 (30.5)	
Same	240 (62.0)	
Increased	29 (7.5)	
Eggs		< 0.001
Decreased	112 (29.0)	
Same	221 (57.3)	
Increased	53 (13.7)	
Dairy		< 0.001
Decreased	100 (26.0)	
Same	267 (69.4)	
Increased	18 (4.7)	
Legumes		< 0.001
Decreased	104 (26.9)	
Same	259 (66.9)	
Increased	24 (6.2)	
Nuts and seeds		< 0.001
Decreased	112 (29.0)	
Same	256 (66.3)	
Increased	18 (4.7)	
Refined grains and baked goods		0.042
Decreased	64 (16.5)	
Same	278 (71.8)	
Increased	45 (11.6)	
Whole grains		0.183
Decreased	43 (11.1)	
Same	312 (80.8)	
Increased	31 (8.0)	
Potatoes and other roots and tubers		< 0.001
Decreased	170 (43.9)	
Same	201 (51.9)	
Increased	16 (4.1)	
Vegetable oils		0.085
Decreased	33 (8.6)	
Same	303 (78.7)	
Increased	49 (12.7)	
Desserts and ice cream and fried foods obtained away from home		< 0.001
Decreased	76 (19.7)	
Same	279 (72.5)	
Increased	30 (7.8)	
Sugar sweetened beverages		< 0.001
Decreased	83 (21.9)	
Same	269 (71.0)	
Increased	27 (7.1)	

*Diet quality is calculated by difference the number of times a specific food was eaten per week between pre-Covid (Q: During the time period before coronavirus, how many days per week did you consume these foods?) vs. during Covid (In the last seven days, how many days did you consume these foods?)

**Wilcoxon Signed Rank Test was run on all variables to compare diet quality between pre-Covid and during Covid

Using the MDD-W calculations, we were able to identify a significant decrease in diet diversity of the communities surveyed with over a 1-point drop in the score pre- (mean = 9.25, SD = 0.05) and post-COVID-19 (mean = 8.19, SD = 0.09), *t*(393) = 13.8, *p* < 0.001. Within the categories used to calculate the MDD-W, we noted that decreases in consumption were most prevalent in legumes (10% decrease), nuts and seeds (17% decrease), dairy (16.3% decrease), and other vitamin A rich fruits and vegetables (which were not dark green leafy vegetables) (21% decrease) (Table 6).

**Table 6:** MDD-W calculation

	Pre-Covid	February 2022	*p value
Grains, Roots and Tubers Include whole grains, refined grains and baked goods, potatoes, other roots and tubers	391 (99.2)	385 (97.7)	0.11
Legumes	378 (95.9)	335 (85.0)	< 0.001
Nuts and seeds	362 (91.9)	295 (74.9)	< 0.001
Dairy	252 (64.0)	188 (47.7)	< 0.001
Meats, poultry and fish Include poultry, red meat, processed meat, fish	388 (98.5)	362 (91.9)	< 0.001
Eggs	375 (95.2)	343 (87.1)	< 0.001
Vit A rich dark vegetables	392 (99.5)	373 (94.7)	0.001
Other Vitamin A rich fruits and vegetables	346 (87.8)	263 (66.8)	< 0.001
Other vegetables Include other vegetables and cruciferous vegetables	394 (100.0)	365 (92.6)	< 0.001
Other fruits Include whole citrus fruits and other whole fruits	365 (92.6)	319 (81.0)	< 0.001
Healthy grains	360 (91.4)	279 (70.8)	< 0.001
MDD-W (mean, SD)	9.25 (1.04)	8.19 (1.76)	< 0.001

*p value based on Wilcoxon signed-rank test

Whole grain, root, and tuber consumption remained relatively stable from pre-covid to post-covid (99.2–97.7%); however, when whole grains alone were separated out (not including refined grains, baked goods, potatoes and other roots and tubers), there was a decrease in consumption from pre-covid to post-covid by 21% (81 households). Using ANOVA testing, we did not find statistical significance in the association between MDD-W change and the correlating change in food prices for staples, pulses or animal sources.

At the end of our questionnaire, when we offered participants the opportunity to provide additional commentary. About one third of participants provided additional comments with most stating a version of:

“*It is difficult to find food because of the lack of work*”

*“Business stopped because of corona”.*

“*Things have really become expensive, hence we can’t afford to buy most items*”

“*Food is hard to find and there is no help with seeds and fertilizer.*”

## Discussion

Overall, we found that diet diversity in Chilanga district decreased during the COVID-19 pandemic and there were high rates of food insecurity in our sample. This was most notable in the decreased consumption of healthy grains, legumes, nuts and seeds, and dairy products. In addition to the drop in diet diversity, there was greater food insecurity during the pandemic. We found that there was an increase in the consumption of refined grains and “unhealthy” foods. These findings are similar to published data from other LMICs. A study in Peru found that while consumption of other goods decreased, the prevalence of sugar‐sweetened beverage consumption remained high in families [15]. Our study also correlates with and builds on the ARISE data which showed that in other sub-Saharan African countries, there was a decrease in dietary diversity [3, 26].

Furthermore, we found an increase in food prices across the board which likely contributed to further food insecurity as noted by respondents. This is consistent with the findings from the ARISE Network, which identified a similar rise in food prices following the start of the pandemic in Burkina Faso, Ethiopia and Nigeria [3]. One study in Sri Lanka also showed that COVID-19 associated drops in income were associated with increased food insecurity [27, 28]. In our population, food insecurity seems to have been driven both by food markets and the loss of jobs. Despite the decrease in food security, there was no significant difference in the assistance received from governments, NGOs, or other organizations after COVID-19. This is a critical finding to direct further intervention given the long-lasting impacts of food insecurity and malnutrition [18]. A recent analysis showed that peri-urban and rural markets within Zambia do not demonstrate the resilience or sustainability to result in a robust recovery in the food and agricultural sectors [8]. This suggests that the impact of the pandemic on food security and nutrition that we identified will be long-lasting and thus require continued monitoring and ensuring adequate attention and intervention.

Zambia was one of the first countries in sub-Saharan Africa to implement a lockdown in March 2020 [2, 29]. While these lockdowns may have been needed to slow the spread of COVID-19, in many low-income countries such as Zambia, they resulted in higher poverty levels, unemployment, and decreased modes of economic survival, particularly for those in the informal sector [29]. In Zimbabwe it was found that the lockdown period was associated with increases in food prices, decreases in diet diversity and disruptions in consumption patterns [30]. Similarly in nine sub-Saharan African countries, this period was associated with a negative impact on the four dimensions of food insecurity (availability, access, utilization, and stability) [28].

The driving factors for decreased dietary diversity are multifactorial; however, in our study population there appeared to be a notable difference in food security after the pandemic with participants noting the impact of economic factors such as food prices as major contributors to changes in food security. This is comparable to findings from the ARISE study as well in which social vulnerability had a negative correlation with diet quality [26]. In February 2021, when this survey was conducted, almost 28% of our sample was unemployed and another 21% self-identified as casual workers. These jobs were the most affected by the pandemic [29]. Using the perspective of the four dimensions of food security, our study also demonstrated that access and stability remain a significant challenge in Zambia [31].

As Zambia returns to a new sense of normalcy and the pandemic subsides, it remains to be seen what long-term impacts will result from this acute period. Currently, there appears to be very little published literature on the impacts of COVID-19 on food security in Zambia or globally at this time, though between 2022 and 2023 the number of food insecure people did increase indicating an upward trend. However, at the time of this writing, it has been less than a year since the WHO declared the end of the COVID-19 pandemic [32] and therefore no true long-term effects have manifested.

### Limitations

While our findings are limited to Chilanga district, our results are only applicable to that of a peri-urban environment and may not be generalizable to more rural communities where resources such as transportation or access to food, education and work may differ and affect food security measures. In addition, our MDD-W was based off of 7-day consumption recall rather than a 24-h recall which may affect the validity and precision of the results [33]. Another notable limitation of our survey methodology was the use of recall for the last seven days as well as well as in the pre-COVID period introducing a recall bias.

### Strengths

The strengths of our study included the adaptation of existing tools which allowed for more direct comparisons. Additionally, we were able to establish a baseline malnutrition prevalence in our population While we were not able to assess rates of malnutrition in the pre-COVID-19 period, there were significant rates of malnutrition peri-COVID-19 across the age groups (details are provided in a separate publication) [19]. The association between food insecurity and malnutrition makes this a critical finding for us to build upon. Having this baseline will allow for longitudinal monitoring of this community over time and measure both health and economic impacts of subsequent interventions. Based on the final question asking participants if there was anything else they wanted to share and respondents focus on job loss and food prices, further qualitative assessments should be pursued to understand changes in the workforce caused by the pandemic and potentials areas for economic development at the community level.

## Conclusion

The COVID-19 pandemic has had significant impacts on food security globally and these impacts have been particularly prominent in vulnerable populations. The economic impacts of the lockdowns imposed to slow the spread of disease have had long-lasting impacts and will likely continue to persist for years to come. Vulnerable populations and those with lower socio-economic status, like much of our study populations are more likely to be impacted by these changes [1]. Our study demonstrated the significant impact of the COVID-19 pandemic on both food security and diet diversity in a predominantly rural district in Zambia supporting the initial hypothesis. These are critical data and must be addressed through increased social safety nets and targeted funding for high-risk individuals. Food security is a crucial component to addressing malnutrition in LMICs and is an area that local and national governments must invest in to maintain healthy and productive populations both in the immediate aftermath of COVID-19 as well as over the long term.

## Data Availability

Not applicable.
